# Real World Data of Laboratory Changes and Immunophenotyping in Patients with Multiple Sclerosis Treated with Ofatumumab—Single Center Experience

**DOI:** 10.3390/biomedicines14030606

**Published:** 2026-03-09

**Authors:** Ružica Gudelj Zorić, Marija Radmilo, Petar Terze, Vana Košta

**Affiliations:** 1 Department of Neurology, University Hospital of Split, 21000 Split, Croatia; rgudelj@kbsplit.hr (R.G.Z.); mradmilo@kbsplit.hr (M.R.); pterze@kbsplit.hr (P.T.); 2 School of Medicine, University of Split, 21000 Split, Croatia

**Keywords:** ofatumumab, multiple sclerosis, anti-CD20 therapy, immunophenotyping, T cells, B cells, natural killer cells, real-world data

## Abstract

**Background/Objectives**: Ofatumumab is a fully human anti-CD20 monoclonal antibody approved for the treatment of relapsing forms of multiple sclerosis (MS). While its efficacy and safety have been demonstrated in clinical trials, real-world data focusing on laboratory changes and detailed immunophenotyping during treatment remain limited. The objective of this study was to assess routine laboratory parameters and immunophenotyping profiles in ofatumumab-treated patients in a real-world setting. **Methods**: We conducted a retrospective, single-center real-world study including 59 patients with relapsing–remitting MS treated with ofatumumab. Routine laboratory parameters were analyzed at the baseline and 6–12 months after treatment initiation. Immunophenotyping by flow cytometry was available for a subset of 29 patients. Infections were assessed during a follow-up period of at least six months. Paired comparisons were performed using the Wilcoxon signed-rank test. **Results**: Ofatumumab induced a profound and sustained depletion of CD19+ B cells (*p* < 0.001). Total T cells, CD4+ and CD8+ T-cell counts, the CD4/CD8 ratio, and natural killer (NK) cells remained largely stable over time. NK cells and helper T cells showed a numerical increase without statistical significance. IgM levels and relative lymphocyte percentages showed a statistically significant decrease compared with baseline (*p* = 0.047 and *p* = 0.016, respectively), while remaining within reference ranges. Other routine laboratory parameters remained stable. Reported infections were infrequent and predominantly mild. **Conclusions**: In this real-world cohort, ofatumumab demonstrated a favorable immunological and laboratory profile consistent with its known mechanism of action. These findings suggest that routine laboratory monitoring is sufficient for most patients, while immunophenotyping may be reserved for selected clinical scenarios. Further prospective studies integrating clinical and radiological outcomes are needed to better define the clinical relevance of these immunological findings.

## 1. Introduction

Multiple sclerosis (MS) is an autoimmune, inflammatory, demyelinating disease of the central nervous system in which T and B lymphocytes play a key role [1,2]. It represents a major neurological condition that mostly affects young adults, with a higher prevalence among women, often leading to disability. In 2020, the worldwide prevalence of this neurodegenerative disease was 2.8 million [3,4,5]. Prevalence in Europe is estimated to be 83 per 100,000 [6,7]. Contemporary insights into the pathogenesis of this autoimmune disease suggest that it arises because of the loss of immune tolerance to self-proteins due to a combination of genetic susceptibility and environmental provocation [8,9].

Emerging evidence over the last few years highlighted the involvement of B cells in the immunopathogenesis of multiple sclerosis. The first groundbreaking piece of evidence was the evolution of rituximab in 2008 [10]. Histopathological studies demonstrated B- and T-cell infiltrates within MS lesions, including perivascular CD20+ B cells and ectopic lymphoid structures, supporting local immune activation within the CNS [11,12].

B-cell functions involved in pathogenesis include antigen presentation to T cells, leading to the auto proliferation of brain-homing T cells, the production of pro-inflammatory cytokines and chemokines, and the release of toxic factors. These combined effects ultimately lead to inflammation and neuronal injury by creating demyelinating lesions [13,14,15].

Natural killer cells (NK) are also considered possibly as an important piece of the puzzle in the immunopathogenesis of MS, but the exact mechanism remains unknown [16].

These discoveries have led to disease-modifying treatments that have markedly slowed disease progression and improved patient outcomes. Three anti-CD20 monoclonal antibodies (mAbs), ocrelizumab, ublituximab, and ofatumumab, are currently approved for clinical use for MS, while rituximab is still being used off-label [10,17,18].

Rituximab, a chimeric anti-CD20 monoclonal antibody, was the first B-cell-depleting therapy used in MS after the HERMES (2008) and OLYMPUS (2009) trials demonstrated reduced inflammatory activity [10,19]. It is administered intravenously and induces B-cell depletion primarily via complement-dependent cytotoxicity and antibody-dependent cellular cytotoxicity [20]. Some studies show moderate, transient reductions in specific T-cell subsets (CD4+ and CD8+) and changes in NK-cell activation/exhaustion profiles [21,22]. Long-term treatment has been associated with reductions in immunoglobulins, particularly IgM, which may contribute to infection risk in some patients [23,24,25].

Ocrelizumab, a humanized anti-CD20 monoclonal antibody approved in 2017 for relapsing and primary progressive MS (OPERA I/II, ORATORIO), is administered intravenously every six months. It induces near-complete peripheral B-cell depletion while other cell pools generally remain stable; the relative percentage of T cells among lymphocytes shows an increase, likely due to B-cell depletion, NK-cell numbers are mostly unchanged, CD8+ T cells may show a slight reduction over time, and CD4+ T cells usually remain at a similar level. Gradual reductions in immunoglobulins, especially IgM and sometimes IgG, have been reported and may be associated with increased infection risk in some cohorts [26,27,28,29,30,31,32,33].

Ublituximab is a glycoengineered anti-CD20 monoclonal antibody approved by the FDA in 2022 for relapsing forms of multiple sclerosis based on the ULTIMATE I and II trials. Similar to other anti-CD20 therapies, it induces rapid B-cell depletion predominantly via enhanced antibody-dependent cellular cytotoxicity and is administered intravenously. Available immunological data indicate effective CD19+ B-cell reduction with generally preserved T- and NK-cell counts and no consistent evidence of severe hypogammaglobulinemia during mid-term follow-up [34].

Ofatumumab represents the first subcutaneous (SC) self-administered fully human anti-CD20 monoclonal antibody FDA (2020) and EMA (2021) approved for RMS based on the ASCLEPIOS I and II trials comparing it with teriflunomide [18,34,35]. Depletion of B cells is its main mechanism of action. Probably thanks to its composition in terms of being a fully human antibody, it has a lower potential to induce antidrug antibodies than other mAbs [17,18,36]. Ofatumumab binds a membrane-proximal CD20 epitope with slower dissociation, resulting in efficient complement-dependent cytotoxicity and dose-dependent B-cell depletion. Compared with rituximab and ocrelizumab, B-cell repletion after treatment interruption appears faster, likely due to its binding characteristics [37,38,39,40,41,42,43].

A small subset of T cells expresses CD20 and may also be affected by anti-CD20 therapy, while overall CD4+/CD8+ counts generally remain within reference ranges [38,44,45,46,47]. Ofatumumab treatment has been associated with stable T- and NK-cell counts with occasional mild numerical changes. It increases control of effector T cells, decreases T-cell autoreactivity whilst reducing peripheral CD20+ T cells, and decreases the migratory capacity of T cells, when compared to healthy controls. Total CD4+/CD8+ T-cell counts, however, remain largely within reference ranges [38,44,47]. A flow-cytometry study in RRMS found that ofatumumab treatment induced a slight increase in the absolute number of NK-cell subpopulations, which may be explained as an interplay between B cells and myeloid cells in the context of CD20 depletion [47]. Clinical studies report preserved IgG levels and modest IgM decline without a clear association with increased infection risk in trial and real-world cohorts [18,38,40,48,49].

Anti-CD20 therapies induce profound B-cell depletion without major effects on other blood cell lines, and routine hepatic and renal laboratory parameters typically remain stable [50,51,52].

The objective of our study was to evaluate changes in immunoglobulins and immunophenotyping parameters in ofatumumab-treated patients in a real-world setting and to assess infection occurrence in routine clinical practice. We also wanted to determine how often these tests are a part of routine practice in our center.

## 2. Materials and Methods

### 2.1. Participants

This retrospective study included 59 adult patients with relapsing–remitting multiple sclerosis treated with ofatumumab, selected from a total of 70 patients treated between 1 January 2022 and 1 March 2025 at the Department of Neurology, University Hospital of Split, Croatia.

Inclusion criteria were age 18–70 years, diagnosis of RRMS according to the 2017 McDonald criteria, and at least 6 months of ofatumumab treatment. Exclusion criteria were other forms of MS, treatment duration shorter than six months, or age outside the predefined range.

Eleven patients were excluded from the analysis: eight due to missing follow-up laboratory data, two because treatment duration was shorter than six months at the time of analysis, and one due to insufficient clinical response requiring a treatment switch. Missing follow-up data were mainly related to laboratory monitoring performed outside our center or patients not yet reaching scheduled control visits at the time of data collection.

The laboratory data, as well as medical records of adult ofatumumab-treated patients diagnosed with RRMS from the Department of Neurology of the University Hospital of Split, were retrospectively evaluated.

### 2.2. Collection of Data and Study Procedure

**Figure 1:**
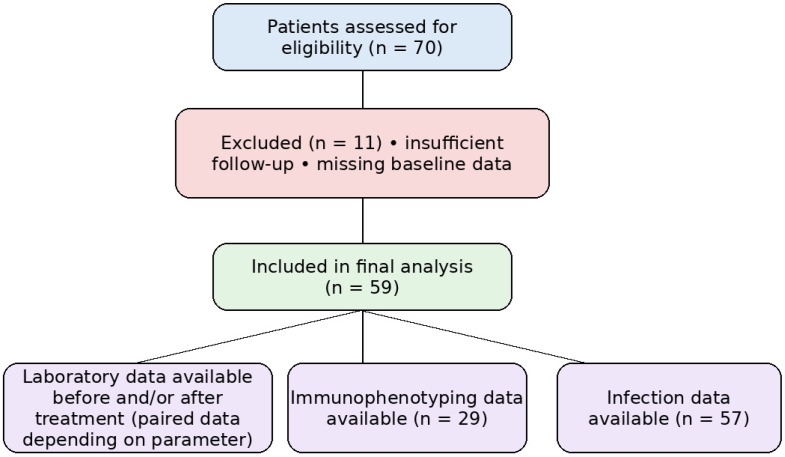
Study flow diagram and data availability.

The collected data included: the demographic and clinical parameters, including sex, the average age of the patients, the average duration of the disease from the year of diagnosis of MS to the year of the first administration of ofatumumab, prior disease-modifying therapy exposure (treatment-naïve vs. previously treated patients), and the occurrence of infections 6 to 12 months after the start of the treatment. Laboratory data included leukocyte counts, relative lymphocyte percentages, liver and renal function tests, and serum IgA, IgM, and IgG levels, as well as immunophenotyping of peripheral blood lymphocytes (total T cells, T-helper cells, cytotoxic/suppressor T cells, CD4+/CD8+ ratio, B cells, and NK cells), assessed up to 3 months before and 6–12 months after the first administration of therapy (Figure 1). Immunophenotyping was performed only in patients in whom flow cytometry was obtained as part of routine clinical care; therefore, paired analyses were conducted in the available subset.

Data on previous and recurring infections regarding our patients were obtained from their medical records. Infections were defined as clinically documented infectious events recorded in medical records. All laboratory parameters were performed in the Central Laboratory of the University Hospital of Split (Department of Medical Laboratory Diagnosis, University Hospital Center Split).

Accordingly, the study was designed as a laboratory-focused observational analysis, and clinical relapse rate, disability outcomes, and MRI activity were not predefined endpoints nor systematically collected.

### 2.3. Statistical Analysis

Statistical analysis was performed using IBM SPSS Statistics for Windows, version 28.0 (IBM Corp., Armonk, NY, USA).

Continuous variables were assessed for normality and are presented as mean ± standard deviation or median with interquartile range (IQR), as appropriate. Categorical variables are presented as absolute numbers N and percentages (%).

For paired comparisons of laboratory parameters and immunophenotyping data before and after ofatumumab initiation, the Wilcoxon signed-rank test was used. Categorical variables were compared using the chi-square test or Fisher’s exact test, as appropriate.

Only patients with available paired pre- and post-treatment measurements were included in comparative analyses; therefore, sample size varied across parameters due to missing data.

All statistical tests were two-sided, and a *p*-value < 0.05 was considered statistically significant.

## 3. Results

### 3.1. Patients’ Characteristics

Seventy patients were screened, of whom 11 were excluded from the study initially due to the exclusion criteria. This study, therefore, included 59 patients, out of which 41 were women (69.5%) and 18 were men (30.5%), treated with ofatumumab. The median age of the subjects (n = 59) at first ofatumumab administration was 41.0 years (IQR 34.5–50.0). The mean disease duration was 5.76 ± 6.15 years before the first ofatumumab administration. Seventeen (28.8%) patients received ofatumumab as the second-line therapy, while previously being treated with interferon-beta, fingolimod, glatiramer acetate, teriflunomide, and dimethyl fumarate. Forty-two (71.2%) patients started ofatumumab treatment as the first-line therapy (Table 1).

Twenty-nine patients had complete paired immunophenotyping data and were included in the lymphocyte subset analysis, while 57 patients’ data were analyzed for infections. The number of patients analyzed for routine laboratory parameters was variable due to the missing data.

### 3.2. Infections

In this cohort, following patients’ treatment with ofatumumab, infections occurred in four patients (7%), while 53 (93%) remained infection-free, corresponding to a low infection rate of 7 per 100 patients during the observed follow-up period. Regarding these subjects, we had medical records for 57 patients due to missing data. No serious cases were recorded; two patients had a moderate COVID infection, one had nasopharyngitis, and one had pyelonephritis.

### 3.3. Routine Laboratory Parameters

All laboratory parameters were within normal reference range before and after six to twelve months of ofatumumab treatment. Nevertheless, we detected a significant decrease in the IgM level and relative lymphocyte percentages within the reference range (*p* = 0.047 and *p* = 0.016, respectively, Wilcoxon Signed-Rank Test, Table 2).

Descriptive statistics for laboratory and immunophenotyping parameters, as well as paired statistical comparisons, are based only on patients with available paired pre-and post-treatment measurements.

### 3.4. Results of Immunophenotyping

**Figure 2:**
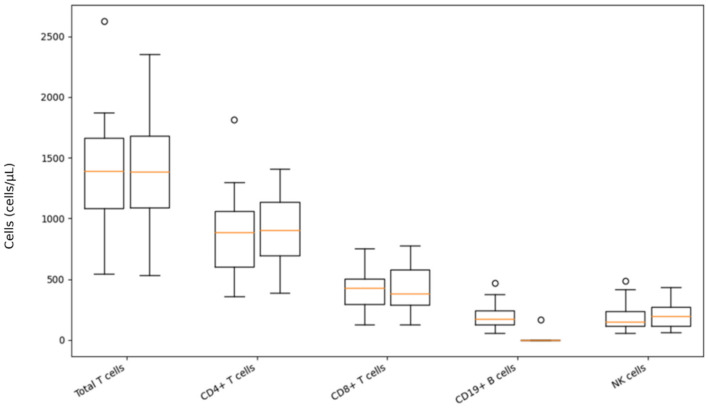
Immunophenotyping data changes after ofatumumab treatment. Note: On each box, the central brown mark indicates the median, and the bottom and top edges of the box indicate the 25th and 75th percentiles, respectively. Whiskers indicate the data range, and circles represent outliers.

The results of immunophenotyping of T-lymphocytes, B-lymphocytes, and NK cells using flow cytometry were analyzed in 29 patients before the first dose and 6 to 12 months after initiation of treatment with ofatumumab. CD19+ B cell count decreased significantly (*p* < 0.001) after ofatumumab therapy. Although total T cells, as well as helper and NK cells, show a tendency to increase, no significant changes occurred regarding their numbers (Table 3, Figure 2).

## 4. Discussion

This retrospective study analyzed a collection of laboratory parameters, including immunophenotyping of peripheral blood lymphocytes in patients with RRMS before administration of the first dose of ofatumumab and at least six to twelve months after the first intake. The occurrence of infections was also monitored by follow-up using patients’ medical records. In our study, as expected, we confirmed a significant depletion of B-lymphocytes (CD19+ B-lymphocytes) after the drug administration. The main study findings confirm no other significant changes in laboratory parameters or immunophenotyping, alongside a very low infection rate (7%) [30,38]. In the era of highly effective disease-modifying therapies, conventional clinical and MRI outcomes may be insufficient to detect subtle but potentially meaningful therapeutic effects. Refining outcome measures to include complementary laboratory and immunological biomarkers has been proposed as an important direction in multiple sclerosis research [53]. This perspective provides context for our focus on immunoglobulins and lymphocyte subsets in a real-world cohort.

We met challenges regarding data collection because some patients were doing routine follow-up outside the hospital, some were non-compliant with scheduled follow-up visits or laboratory testing, and for some, regular laboratory testing was not even recommended. As relative lymphocyte percentages are commonly used for longitudinal monitoring in routine clinical practice, they were chosen for this retrospective analysis. All available laboratory and immunophenotyping data were analyzed, with paired pre- and post-treatment comparisons restricted to patients with available paired measurements. Although follow-up intervals were not uniform across patients, the observed laboratory and immunological changes were consistent with the expected biological effects of ofatumumab [35,54,55].

In our cohort, patients initiated ofatumumab relatively early after diagnosis (mean 5.76 years), and a high proportion were treatment-naïve (71.2%). The predominance of female patients and the younger adult age profile observed in our cohort are in line with the known epidemiological characteristics of this disease [3,5,7]. Compared with other real-world cohorts reporting lower proportions of treatment-naïve patients [56,57], this reflects differences in treatment strategies across centers and suggests early real-world use of a high-efficacy therapy in our routine clinical practice.

The infection rate in our cohort was low (7%), and no serious infections were recorded. These findings are consistent with ASCLEPIOS and ALITHIOS data reporting low serious infection rates [18,40,49,58]. It should, however, be noted that due to the retrospective character of this study, it is possible that not all of the cases were documented in the medical records. In big real-world cohorts, the number of more serious and recurrent infections was greater in patients treated with rituximab or ocrelizumab. This may be related to a lower tendency for hypogammaglobulinemia with ofatumumab treatment, especially when it comes to IgM levels [25,31,59,60].

Serum IgM declined significantly but remained within reference ranges, while IgG and IgA levels remained stable. It is important to note that the reduction of IgM was not followed by an increased rate of infections, as reported earlier. This pattern is consistent with phase 3 and extension data showing preserved IgG and modest IgM reduction without clear association with infection risk [18,38,40,49]. In contrast, more pronounced and cumulative reductions in IgM and, in some cohorts, IgG have been described, which may be linked to higher infection rates when it comes to other mAbs. The observed IgM reduction may reflect selective depletion of CD20+ naïve and memory B cells, while long-lived plasma cells responsible for IgG and IgA production remain preserved. Although subcutaneous administration and pharmacodynamic differences may contribute to this pattern, direct comparisons between therapies are limited by differences in study design [24,25,31,59,60]. In addition to immunoglobulin changes, only minimal alterations in routine laboratory parameters were observed. Relative lymphocyte percentages showed a statistically significant decline but remained within reference ranges, and no clinically meaningful leukopenia was detected. Although this change reached statistical significance, its clinical relevance is limited, as relative lymphocyte values may be influenced by variations in total leukocyte counts. Ocrelizumab and rituximab, on the other hand, have been more frequently associated with cumulative cytopenias and, therefore, higher infection burden in certain real-world series [24,25,31,59]. Renal and hepatic parameters (creatinine, urea, AST, ALT, and GGT) remained stable, in line with long-term extension data from the ALITHIOS program [30,40,50,51,52]. Overall, these findings are consistent with the established laboratory safety profile of ofatumumab reported in larger clinical and real-world studies [61,62].

Immunophenotyping in a subset of patients showed the expected, marked depletion of CD19 B cells after ofatumumab treatment. Total T lymphocytes, CD4 helper, CD8 cytotoxic, and NK cells remained numerically stable, with only mild and non-significant trends toward an increase in helper T and NK subsets. No statistically significant shifts in the CD4/CD8 ratio were observed. This profile is in line with flow-cytometry studies demonstrating that ofatumumab predominantly targets CD20-positive B cells, while overall T-cell and NK-cell counts remain largely preserved, without evidence of broad T-cell lymphopenia [30,44,47,63]. The observation that some T-cell and NK-cell parameters in this cohort show a slight upward trend may reflect a relative redistribution and recovery of non-B lymphocyte compartments in the context of sustained B-cell depletion. This phenomenon is also reported in other ofatumumab and ocrelizumab cohorts where CD3 counts remain stable, but the proportion of T and NK cells among lymphocytes appears increased [47]. Experimental studies suggest that ofatumumab shifts T-cell subsets towards a more regulated pattern, which is even seen as a favorable adjustment of the T-cell response. Reported data do not show a link between such mild increases in NK cells or specific T-cell subsets and a higher infection rate [38,47,63,64]. Similar immunophenotyping data have been reported for ublituximab, where more frequent early sampling revealed transient shifts in memory T-cell subsets and NK-cell counts during the first weeks to months after treatment initiation. As immunophenotyping in our study was performed at 6–12 months, we may have missed such early dynamic changes and instead captured more stable, mid-term immune patterns, as also suggested in longitudinal anti-CD20 cohorts [63,65,66].

Together with the profound CD19 reduction, these findings support the concept that ofatumumab exerts its clinical effects mainly through selective B-cell depletion and modulation of pathogenic T-cell activity, while mostly sparing other immune cell pools. This evidence may help in further explanation regarding the low serious infection rates compared with some other high-efficacy DMTs that induce more widespread immune suppression [30,38,60].

Finally, it is necessary to emphasize that immunophenotyping in our cohort was available for only 29 patients, which reflects the fact that routine flow cytometry monitoring is not yet standard practice for ofatumumab at our center. This is in accordance with the current recommendations by EMA SmPC for ofatumumab that primarily focus on clinical assessment, blood counts, and immunoglobulin levels rather than mandatory flow cytometry for every patient. In addition, routine laboratory assessments were not available for all patients and were not obtained at strictly predefined time points. This case reflects the variability of follow-up inherent to real-world clinical practice. Nevertheless, the observed overall laboratory findings were consistent with the expected safety profile and biological effects of ofatumumab [67,68].

The real-world data of this study provides insight into the laboratory changes and safety of ofatumumab use in routine daily clinical practice. In addition, the single-center design allowed for standardized laboratory testing and consistent longitudinal monitoring across all patients. However, several limitations should be acknowledged. The retrospective design and single-center setting may limit generalizability, while the relatively small sample size, incomplete data, and short follow-up period limit conclusions about long-term outcomes. Furthermore, immunophenotyping was not performed at predefined early timepoints, which may have limited our ability to detect transient immune changes occurring shortly after treatment initiation.

## 5. Conclusions

In this real-world cohort, ofatumumab treatment was associated with the expected depletion of CD19+ B cells and only minimal changes in other lymphocyte subsets and routine laboratory parameters over a relatively short follow-up period. The slight upward tendency observed in T-helper and NK cells, together with marked B-cell depletion, may reflect redistribution within the lymphocyte pool following B-cell removal rather than a true expansion of these populations. Immunoglobulin levels largely remained within reference ranges, and infections were infrequent and mild.

These findings appear consistent with the previously described favorable safety profile of ofatumumab; however, given the retrospective design, limited sample size, and absence of systematic clinical and MRI outcomes, they should be interpreted as supportive laboratory observations rather than proof of safety.

Our results suggest that routine laboratory monitoring is generally sufficient in everyday clinical practice, while extended immunophenotyping may be reserved for selected clinical situations. Further prospective studies integrating clinical and radiological outcomes are needed to better define the clinical relevance of these immunological findings.

## Figures and Tables

**Table 1:** Patient characteristics at baseline.

Parameter	Value
Total patients	59
Age, years	41.0 (IQR 34.5–50.0)
Disease duration, years	5.76 ± 6.15
First-line therapy (DMT-naïve)	71.2% (n = 42)
Second-line therapy (prior DMT)	28.8% (n = 17)
Female sex	69.5% (n = 41)
Male sex	30.5% (n = 18)

DMT-disease modifying therapies.

**Table 2:** Routine laboratory parameters before and after ofatumumab treatment.

Parameter	n (Paired)	Before—Median (IQR)	After—Median (IQR)	*p*
Leukocytes (10^9^/L)	49	6.2 (5.3–7.4)	6.3 (5.1–7.9)	0.302
Lymphocytes (%)	49	28.9 (26.2–36.1)	27.8 (21.5–32.2)	0.016 *
Urea (mmol/L)	49	5 (3.9–5.6)	4.3 (3.7–5.6)	0.103
Creatinine (µmol/L)	48	69 (58.5–78)	69.5 (60–75.5)	0.373
AST (U/L)	48	19 (15.5–23.5)	21 (18–24.3)	0.085
ALT (U/L)	48	22 (17–28)	23 (17–31)	0.334
GGT (U/L)	48	14 (11–21.5)	14 (11–23)	0.592
IgA (g/L)	40	2.2 (1.5–2.7)	2.3 (1.5–2.9)	0.233
IgG (g/L)	45	10.4 (8.6–12.1)	10.5 (8.9–11.9)	0.972
IgM (g/L)	45	1.2 (0.8–1.5)	0.87 (0.7–1.3)	0.047 *

* Wilcoxon signed-rank test. AST = aspartate aminotransferase; ALT = alanine aminotransferase; GGT = gamma-glutamyl transferase, IgA = immunoglobulin A; IgG = immunoglobulin G; IgM = immunoglobulin M. N varies by parameter due to missing data.

**Table 3:** Immunophenotyping parameters before vs. after 6–12 months of ofatumumab therapy.

Parameter	n (Paired)	Before—Median (IQR)	After—Median (IQR)	*p*
Total T cells (/µL)	29	1364 (1063–1671)	1387 (1229–1683)	0.323
CD4+ T cells (/µL)	29	821 (604–1062)	901 (700–1060)	0.219
CD8+ T cells (/µL)	29	442 (320.5–536.8)	396 (327–580)	0.452
CD4/CD8 ratio	29	1.8 (1.53–2.28)	2.0 (1.6–2.8)	0.090
CD19+ B cells (/µL)	29	168.5 (126.3–242.5)	0.0 (0.0–1.0)	<0.001 *
NK cells (/µL)	29	167.0 (115.8–233.8)	209.0 (164.0–314.0)	0.156

* Wilcoxon signed-rank test, two-sided (n = 29).

## Data Availability

The data presented in this study are available on request from the corresponding author due to ethical restrictions and the protection of patient confidentiality.
